# Supplementary material for: Helmet Shape and Phylogeography of the Treehopper Membracis mexicana
Source: Insects. 2023 Aug 14;14(8):704. doi: 10.3390/insects14080704 (PMC10455800; doi:10.3390/insects14080704)
Supplement: Supplementary file 1 [file insects-14-00704-s001.zip › insects-2529639-supplementary.pdf]

Supplementary material

Helmet shape and phylogeography of the treehopper *Membracis mexicana.*

Tables and figures included:

Table S1 Landmark definition.
Table S2 Variation of genetic markers.
Table S3 Number and frequency of haplotypes.
Figure S1 Landmark position.
Figure S2 Centroid size of helmet by host plant.
Figure S3 PCA colored by population.
Figure S4 PCA colored by host plant.
Figure S5 CVA using host plant as classifier variable.
Figure S6 Mitochondrial COI gene haplotype network.
Figure S7 Nuclear WG gene haplotype network.
Figure S8 Nuclear 28S gene haplotype network.
Figure S9 Nuclear H2A gene haplotype network.
Figure S10 Nuclear H3 gene haplotype network.
Figure S11 Geographic distribution and frequency of COI haplotypes.
Figure S12 Geographic distribution and frequency of WG haplotypes.
Figure S13 Geographic distribution and frequency of 28S haplotypes.
Figure S14 Geographic distribution and frequency of H2A haplotypes.
Figure S15 Geographic distribution and frequency of H3 haplotypes.
Figure S16 Bayesian phylogeny using COI.
Figure S17 Bayesian phylogeny using WG.
Figure S18 Bayesian phylogeny using 28S gene segment.
Figure S19 Bayesian phylogeny using H2A gene segment.
Figure S20 Bayesian phylogeny using H3 gene segment.
Figure S21 Skyline plot using mitochondrial (A) and nuclear markers (B).

Table S1. Landmark definition of *Membracis mexicana* in Mexico.

ID	Landmark definition
1	Eye spot lower right margin
2	Eye spot lower left margin
3	Helment base frontal top
4	Anterior edge of the frontal spot
5	Posterior edge of the frontal spot
6	Anterior edge of the dorsal spot
7	Posterior edge of the dorsal spot
8	Anterior-dorsal edge of the caudal girdle
9	Posterior-dorsal edge of the caudal girdle
10	Anterior-dorsal edge of the ending spot
11	Tip of the ending spot
12	Anterior-ventral edge of the ending spot
13	Posterior-ventral edge of the caudal girdle
14	Anterior-ventral edge of the caudal girdle
15	Base of the side spot
16	Upper base of the wing
17	Bottom base of the wing
18	Right margin of the helmet at head
19	Left margin of the helmet at head

Table S2. Variation of genetic markers and neutrality tests, for the total number of insects, collected of *M. mexicana* in Mexico. * and ** are values statistically significant.

	28s	H3	H2A	Wg	COI
**Polymorphic Sites**					
**Number of sequences:**	189	177	190	147	141
**Number of sites:**	660	300	279	275	369
**Segregating sites (S)**	110	19	29	24	72
**Number of haplotypes, h:**	28	22	33	30	54
**Haplotype (gene) diversity, Hd:**	0.797	0.625	0.425	0.874	0.881
**Nucleotide diversity, Pi**	0.01071	0.00342	0.00375	0.03121	0.01807
**Theta (per site) from S, Theta-W:**	0.02879	0.01112	0.01785	0.01568	0.03543
**Fu and Li's Tests**					
**Fu and Li's F* test statistic:**	-6.41793	-3.86732	-4.04503	2.36933	-2.85538
**Statistical significance:**	**, P < 0.02	**, P < 0.02	**, P < 0.02	**, P < 0.02	*, P < 0.05
**Tajima's Test**					
**Tajima's D:**	-2.18358	-1.97102	-2.34419	2.81039	-1.72698
**Statistical significance:**	**, P < 0.01	*, P < 0.05	**, P < 0.01	**, P < 0.01	0.10 > P > 0.05

Table S3. Number and frequency of haplotypes of each gene, the most frequent are highlighted with blue color.

28s	Frecuency	H2A	Frecuency	H3	Frecuency	Wg	Frecuency	COI	Frecuency	COI	Frecuency
1	1	1	144	1	1	1	1	1	10	34	1
2	1	2	1	2	84	2	3	2	1	35	1
3	1	3	1	3	1	3	13	3	1	36	1
4	1	4	1	4	1	4	7	4	1	37	3
5	1	5	1	5	1	5	5	5	1	38	1
6	1	6	1	6	2	6	1	6	1	39	1
7	1	7	1	7	1	7	9	7	1	40	1
8	1	8	6	8	1	8	3	8	1	41	2
9	1	9	1	9	1	9	3	9	2	42	1
10	8	10	1	10	1	10	2	10	1	43	1
11	22	11	1	11	1	11	6	11	1	44	46
12	1	12	1	12	69	12	2	12	3	45	5

13	54	13	1	13	1	13	10	13	1	46	2
14	17	14	1	14	1	14	3	14	1	47	4
15	1	15	2	15	1	15	2	15	1	48	1
16	1	16	1	16	1	16	2	16	1	49	1
17	1	17	1	17	1	17	6	17	1	50	1
18	1	18	2	18	1	18	2	18	1	51	1
19	60	19	1	19	2	19	1	19	1	52	1
20	1	20	1	20	3	20	1	20	1	53	1
21	1	21	1	21	1	21	1	21	1	54	2
22	1	22	2	22	1	22	2	22	1		
23	3	23	1			23	1	23	1		
24	1	24	2			24	1	24	1		
25	1	25	1			25	1	25	1		
26	3	26	6			26	1	26	6		
27	2	27	1			27	47	27	1		
28	1	28	1			28	9	28	1		
		29	1			29	1	29	10		
		30	1			30	1	30	1		
		31	1					31	5		
		32	1					32	1		
		33	1					33	1		

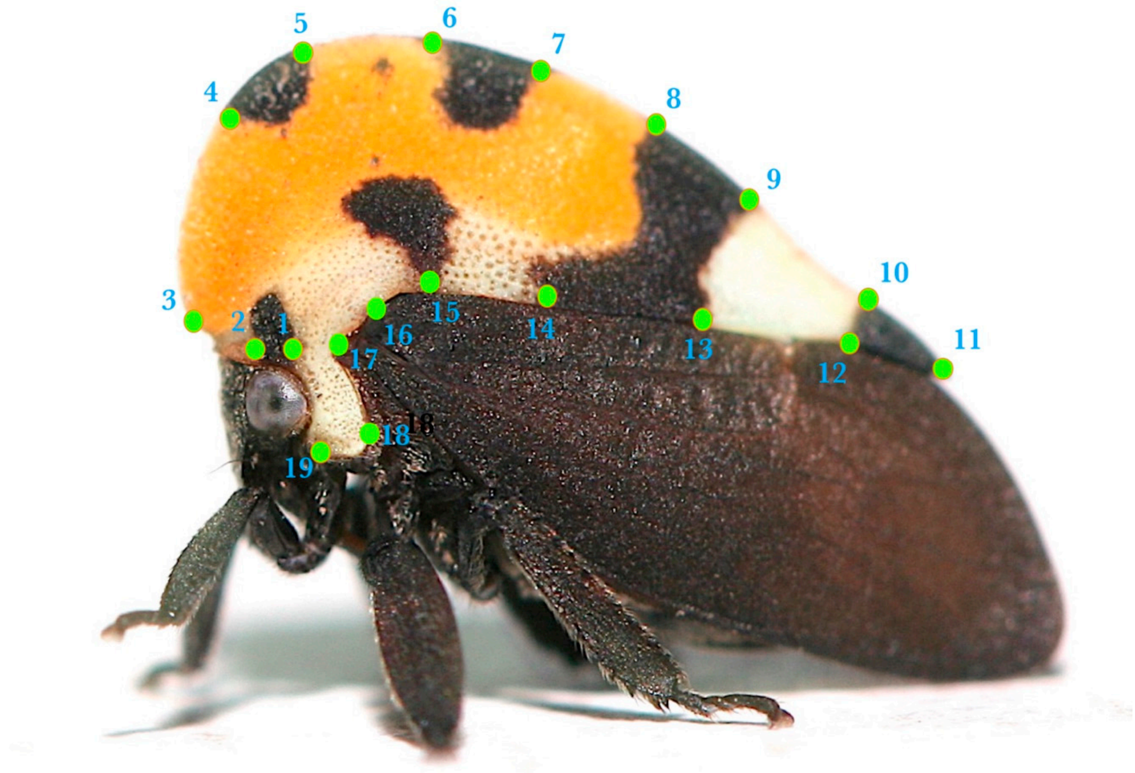

Figure S1. Landmark position at the helmet of *Membracis mexicana.*

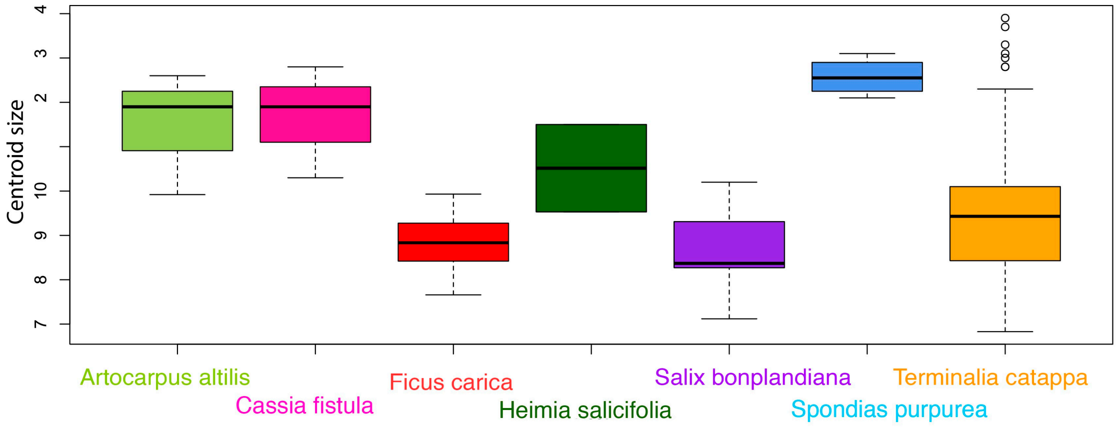

Figure S2. Centroid size of the helmet of *Membracis mexicana* by host plant.

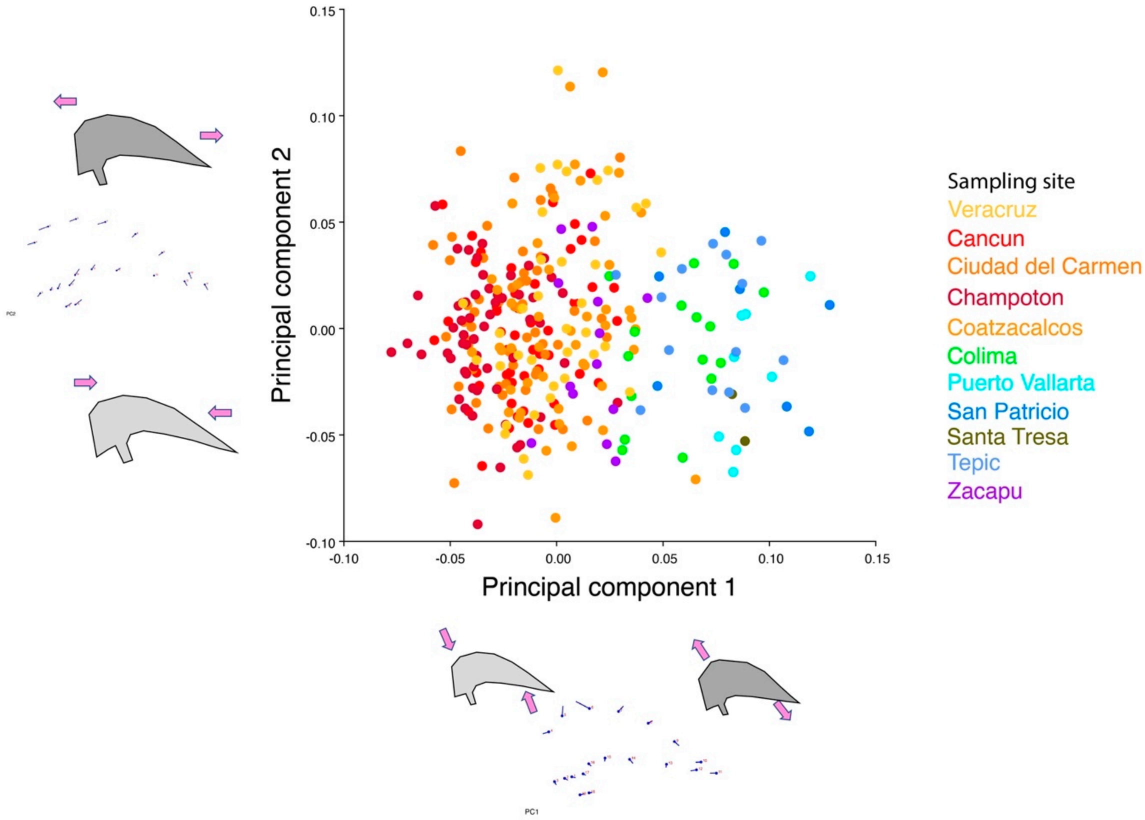

Figure S3. PCA of *Membracis mexicana* colored by population.

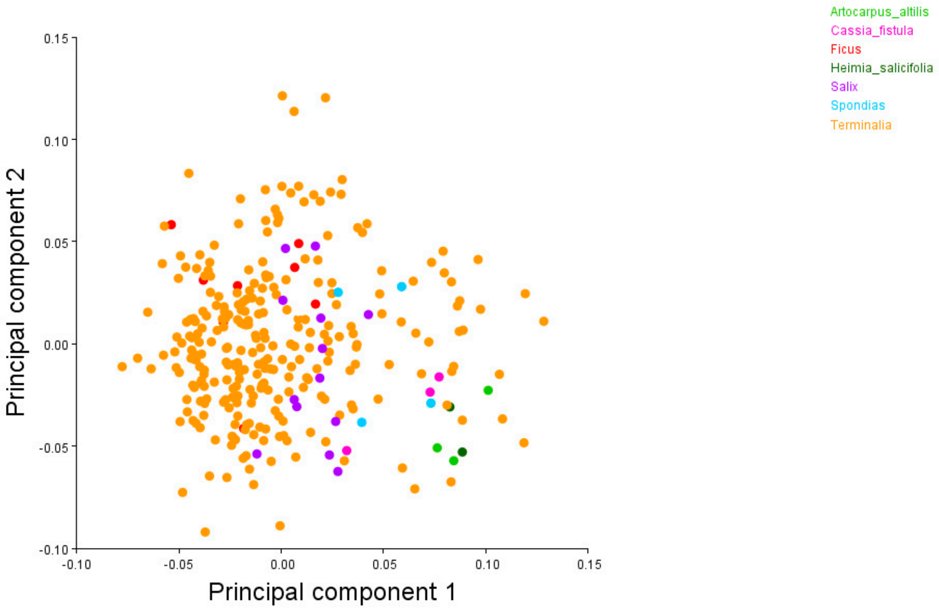

Figure S4. PCA of *Membracis mexicana* colored by host plant.

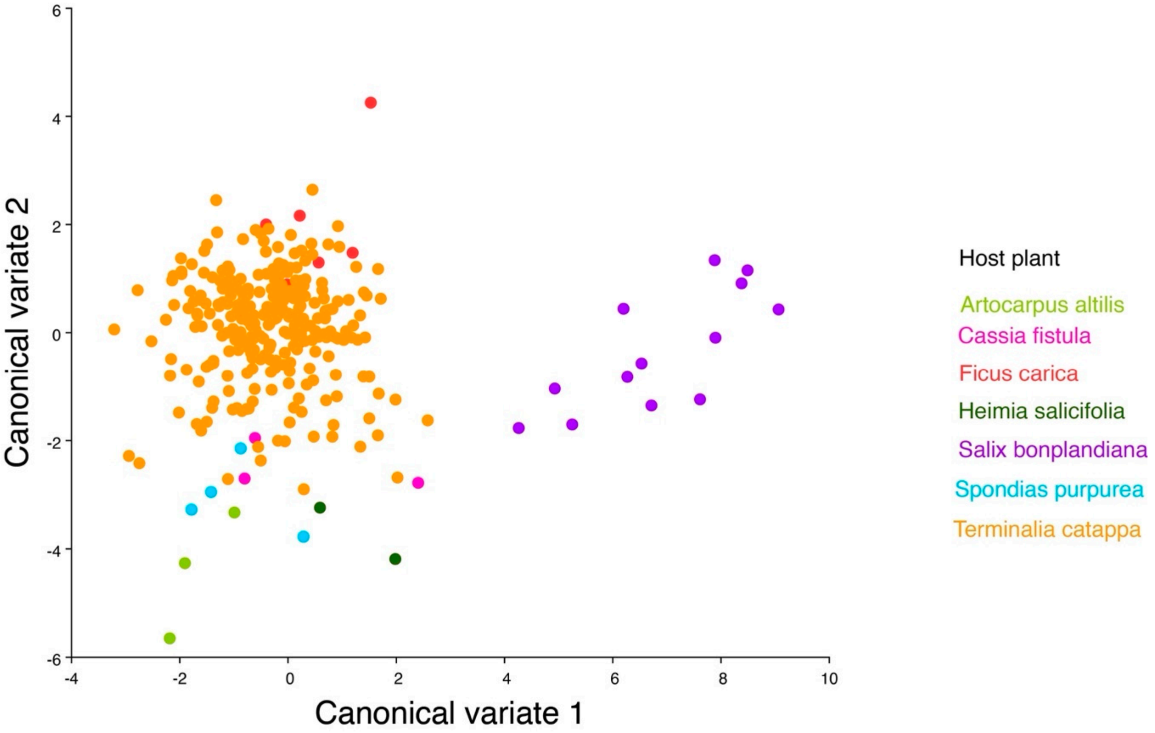

Figure S5. CVA of *Membracis mexicana* using host plant as classifier variable

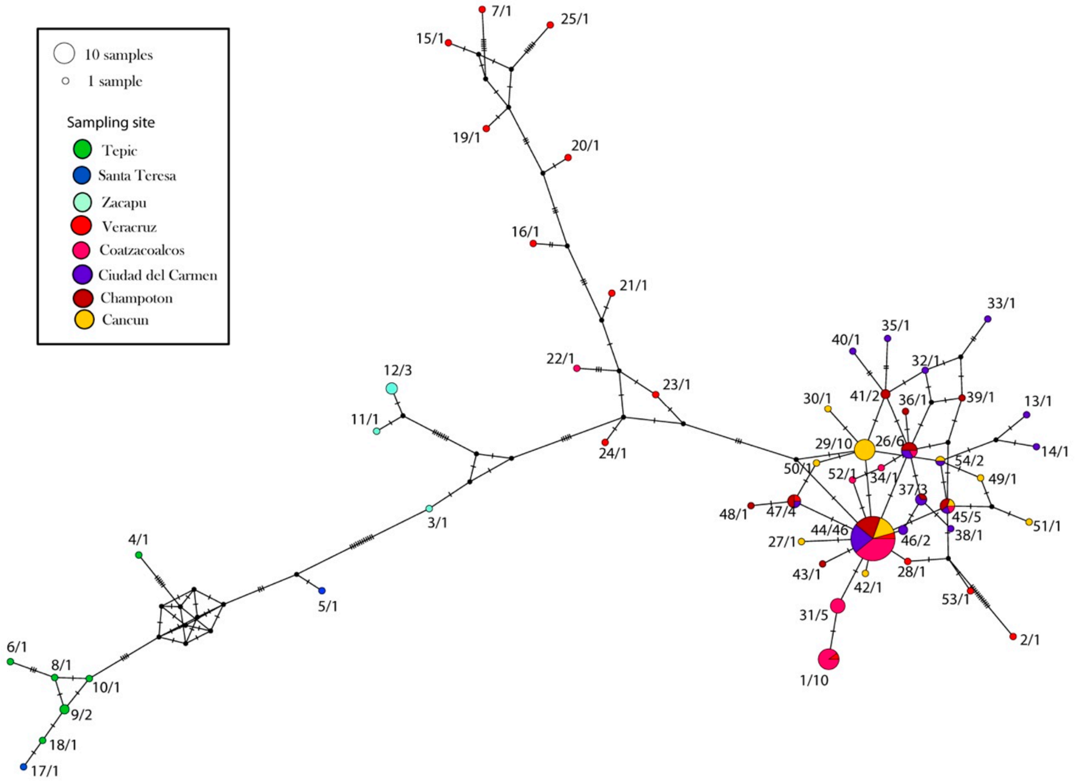

Figure S6. Mitochondrial COI gene haplotype network of *Membracis mexicana.* The circle's size is the haplotype's frequency, which is colored by the population where it was sampled.

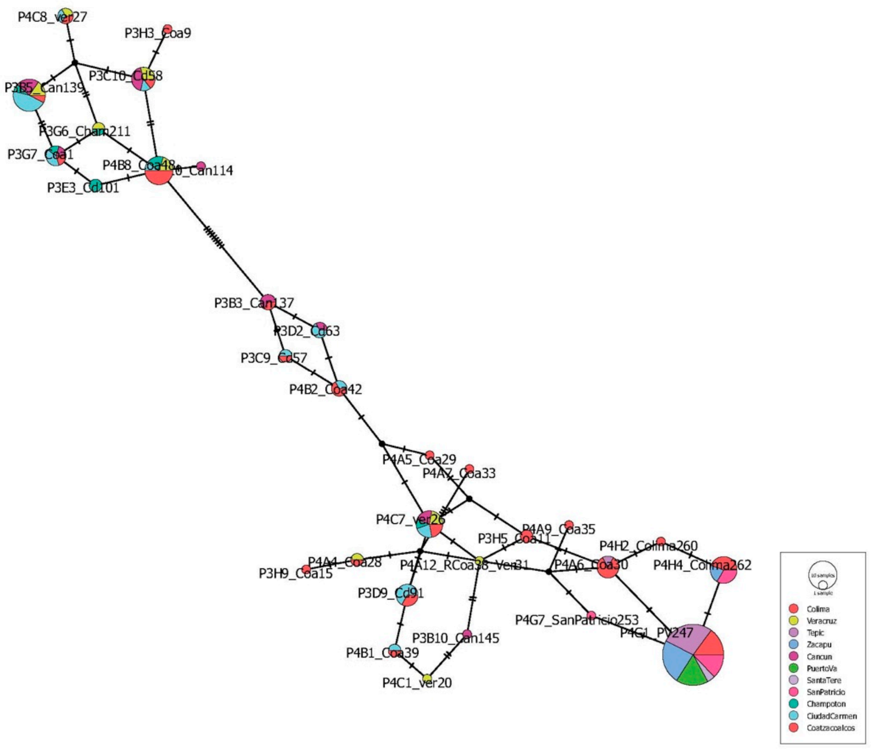

Figure S7. Nuclear WG gene haplotype network of *Membracis mexicana.* The circle's size is the haplotype's frequency, which is colored by the population where it was sampled.

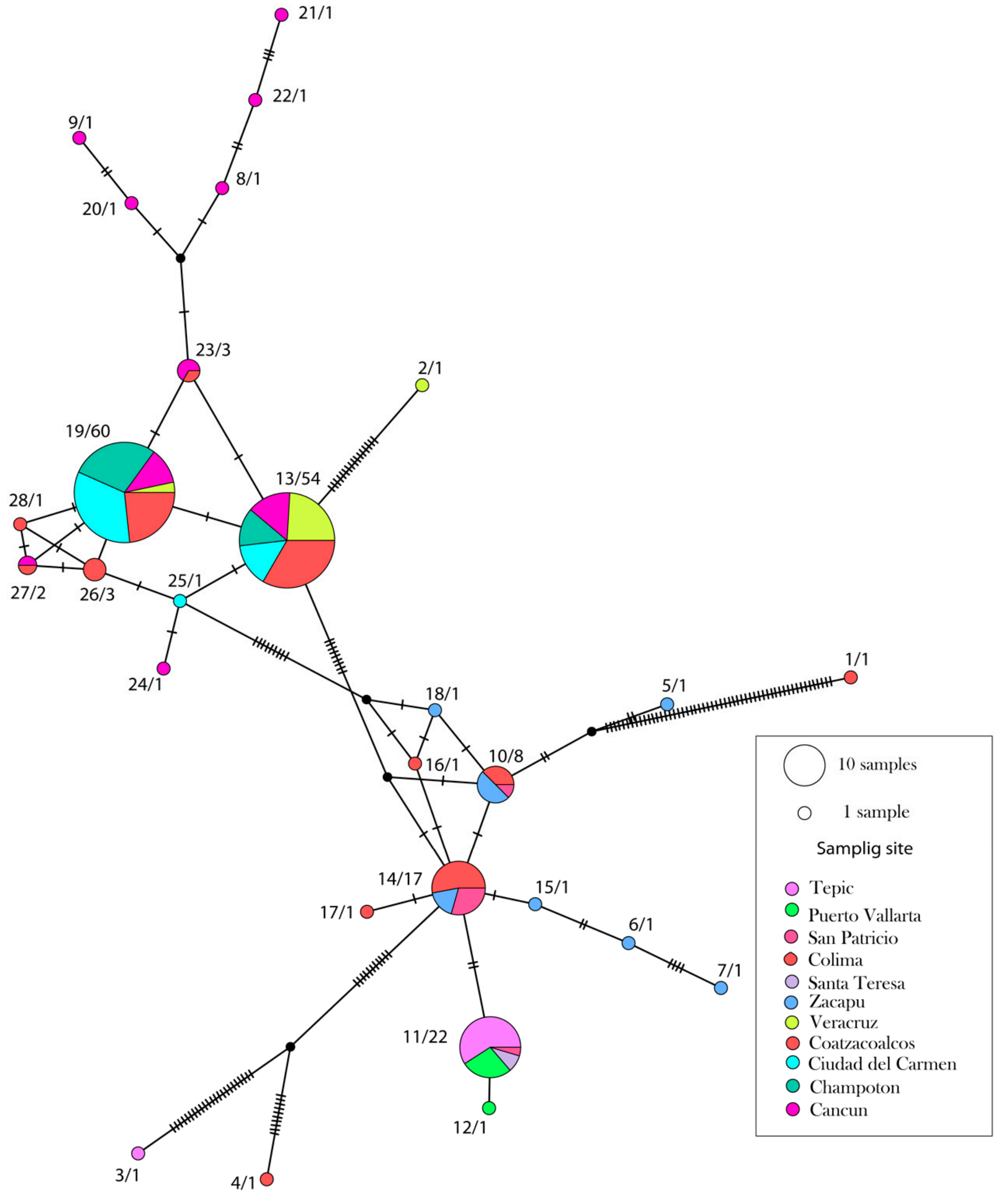

Figure S8. Nuclear 28S gene haplotype network of *Membracis mexicana.* The circle's size is the haplotype's frequency, which is colored by the population where it was sampled.

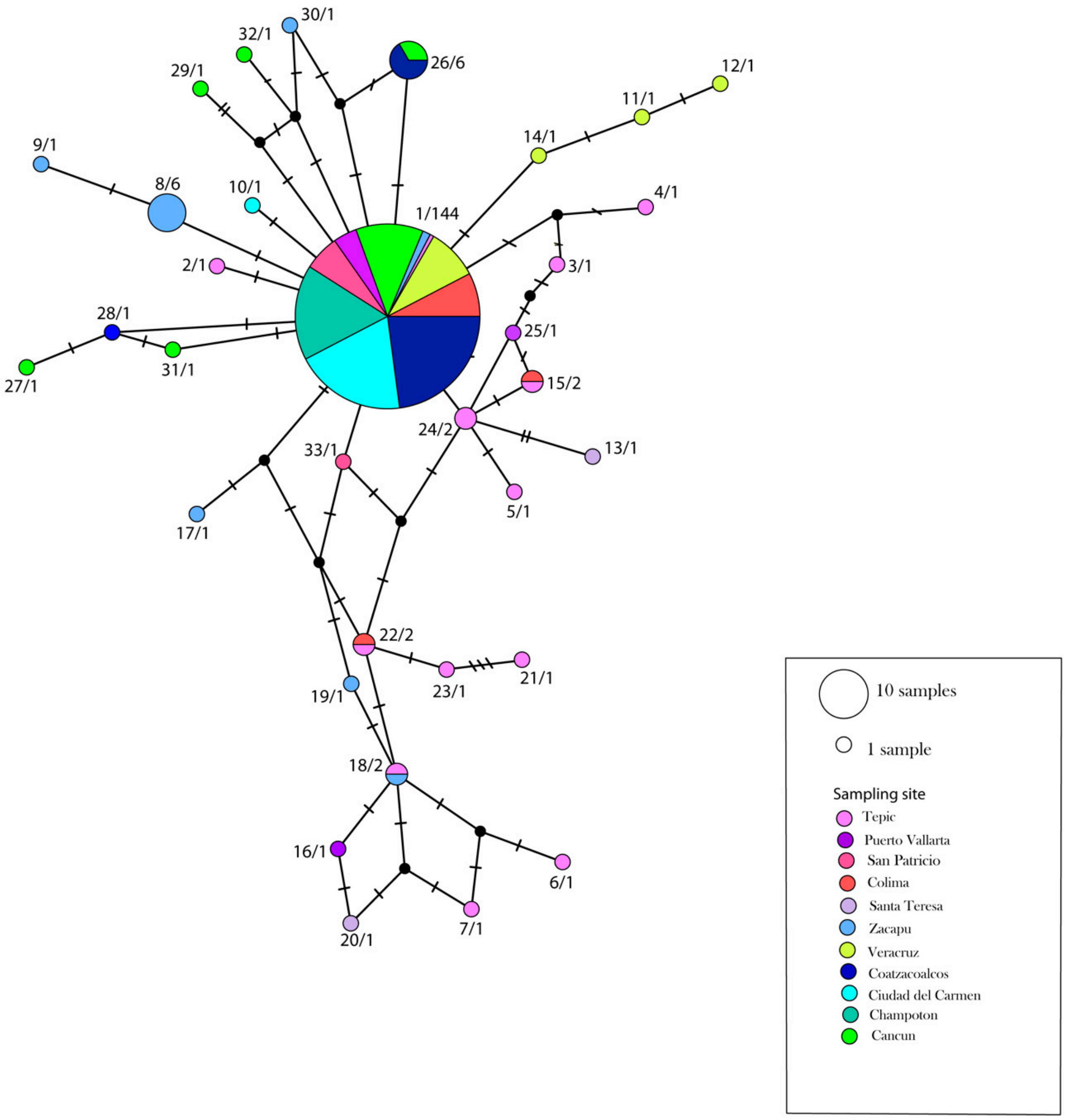

Figure S9. Nuclear H2A gene haplotype network of *Membracis mexicana.* The circle's size is the haplotype's frequency, which is colored by the population where it was sampled.

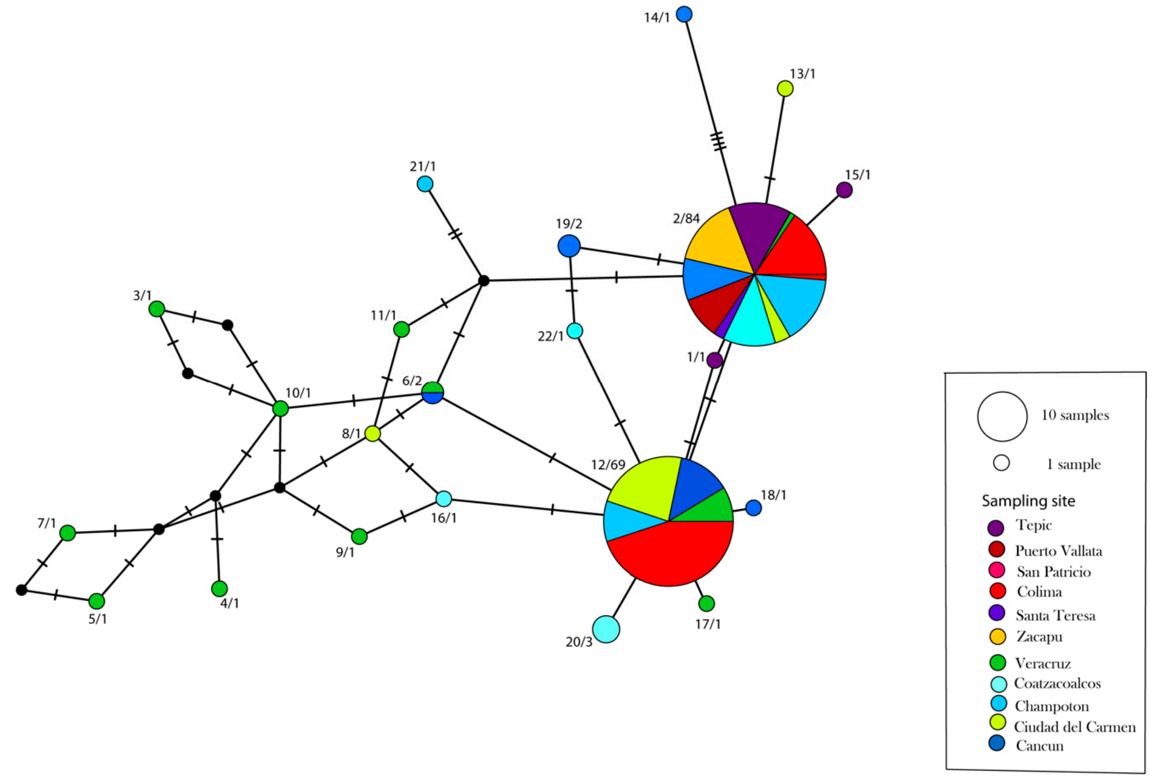

Figure S10. Nuclear H3 gene haplotype network of *Membracis mexicana.* The circle's size is the haplotype's frequency, which is colored by the population where it was sampled.

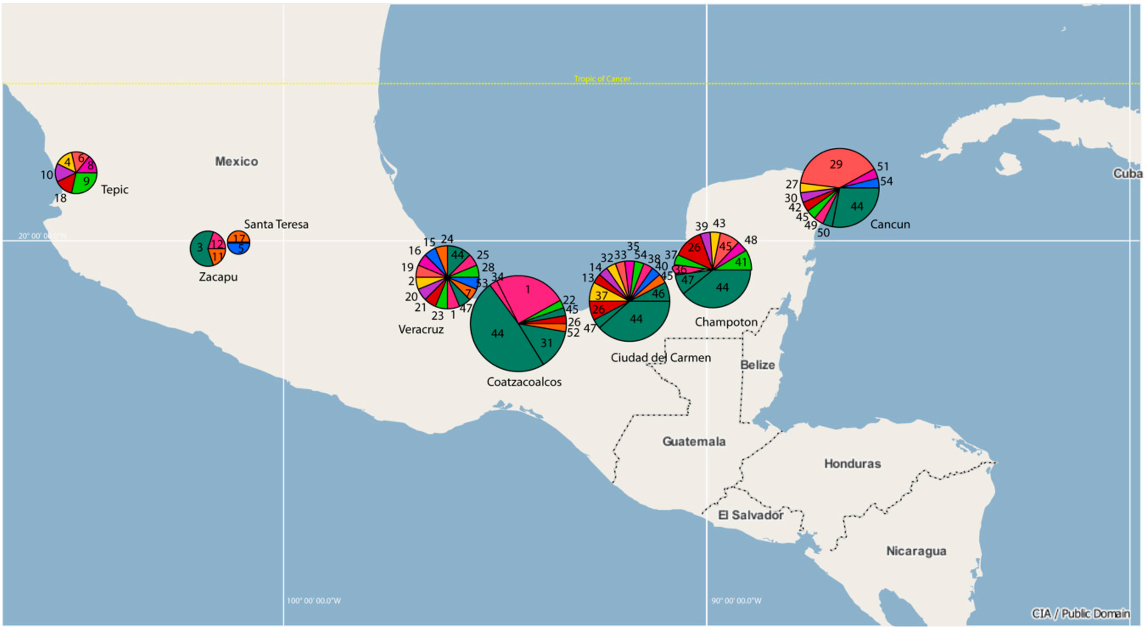

Figure S11. Geographic distribution and frequency of the haplotypes of COI gene of *Membracis mexicana.*

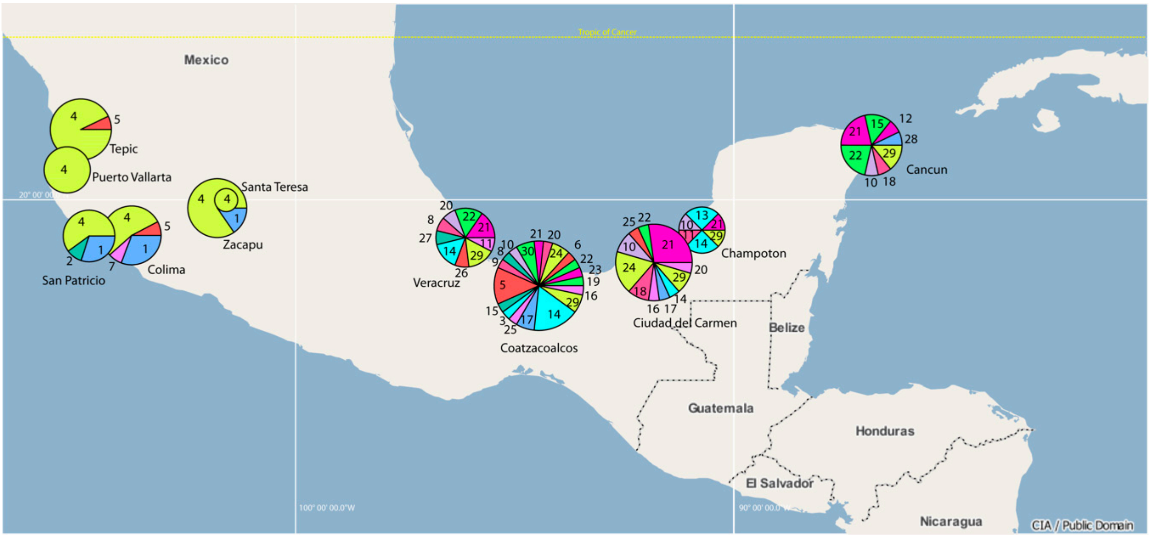

Figure S12. Geographic distribution and frequency of the haplotypes of WG gene of *Membracis mexicana.*

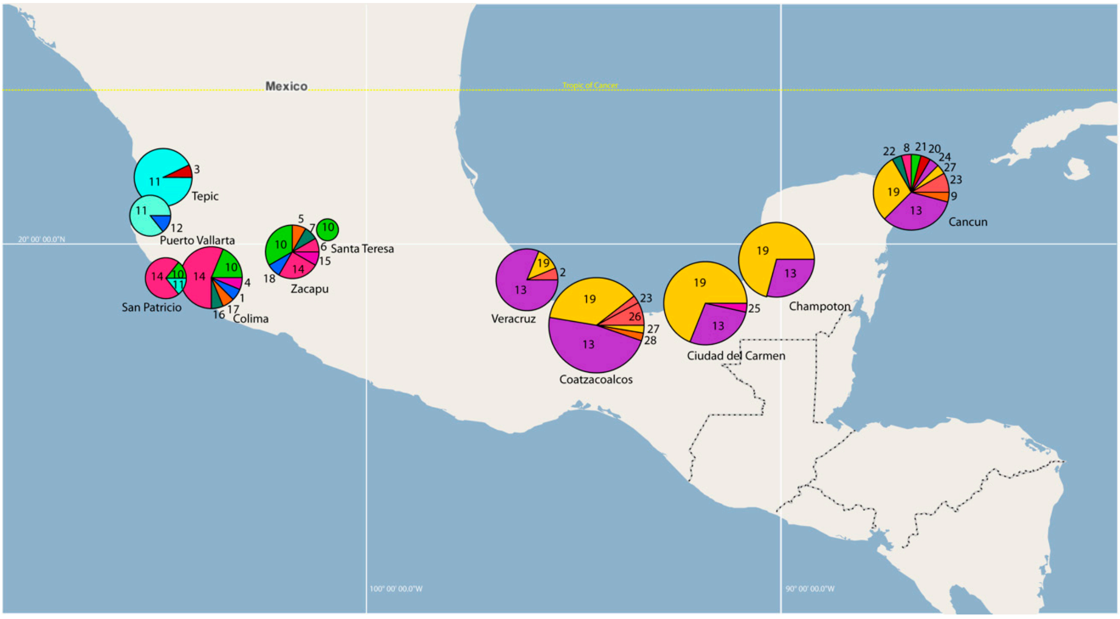

Figure S13. Geographic distribution and frequency of the haplotypes of 28S gene of *Membracis mexicana.*

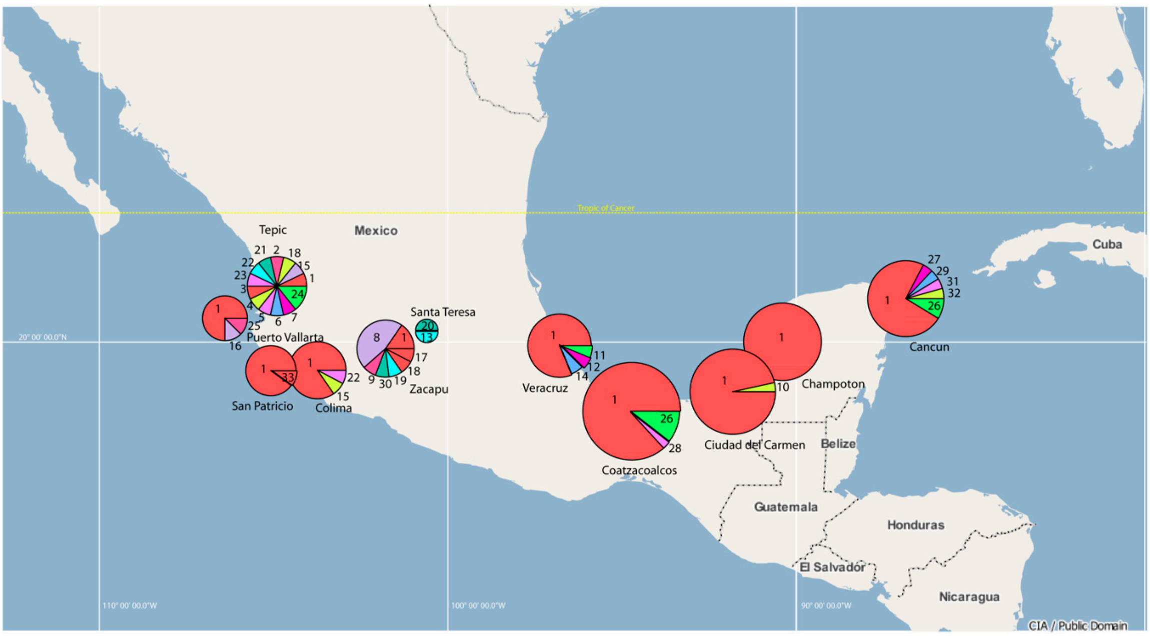

Figure S14. Geographic distribution and frequency of the haplotypes of H2A gene of *Membracis mexicana.*

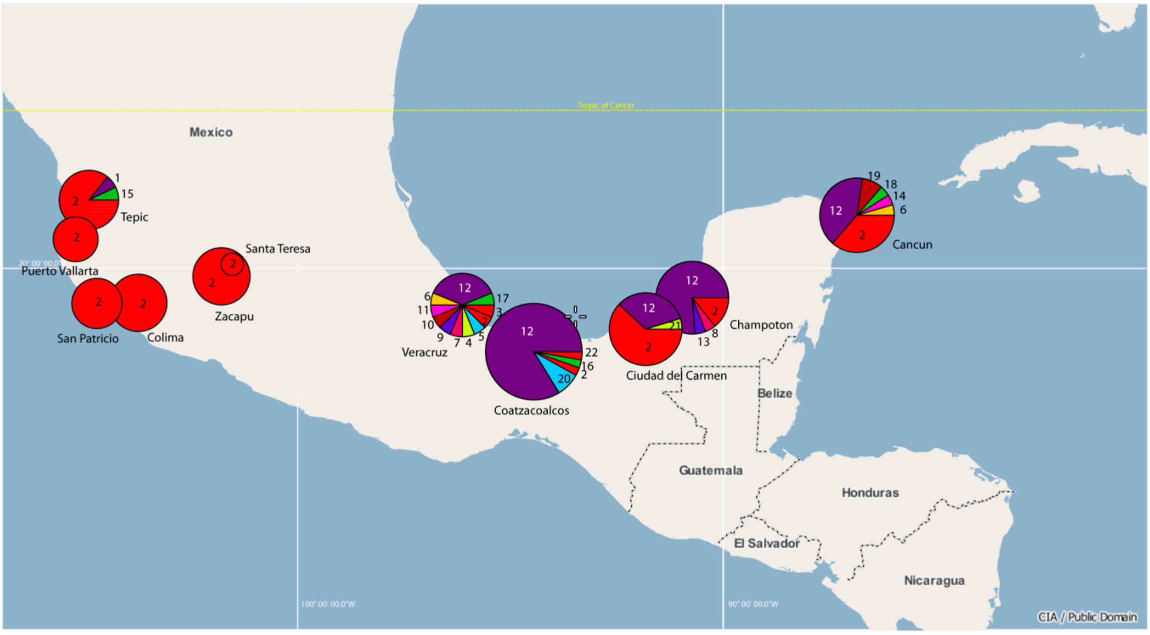

Figure S15. Geographic distribution and frequency of the haplotypes of H3 gene of *Membracis mexicana.*

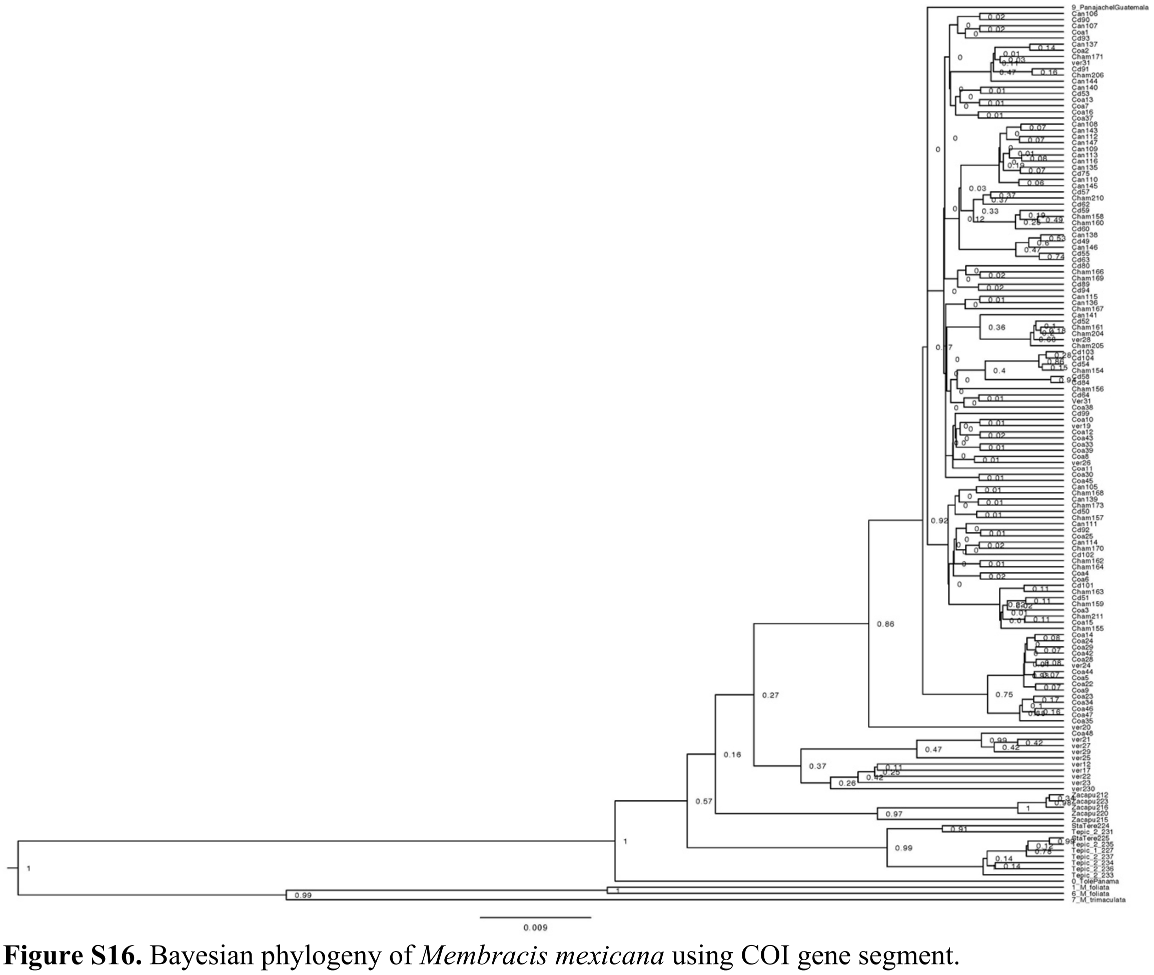

Figure S16. Bayesian phylogeny of *Membracis mexicana* using COI gene segment.

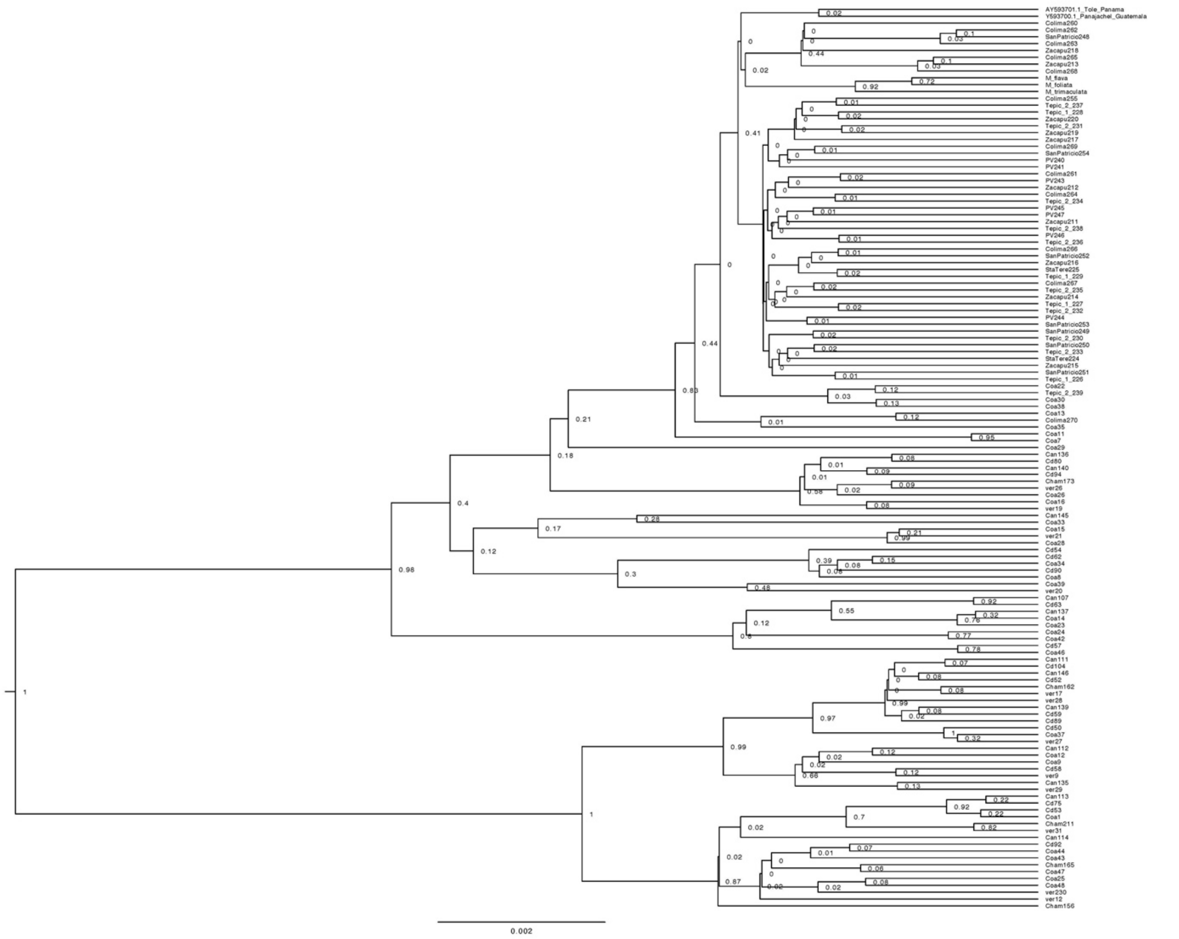

Figure S17. Bayesian phylogeny of *Membracis mexicana* using WG gene segment.

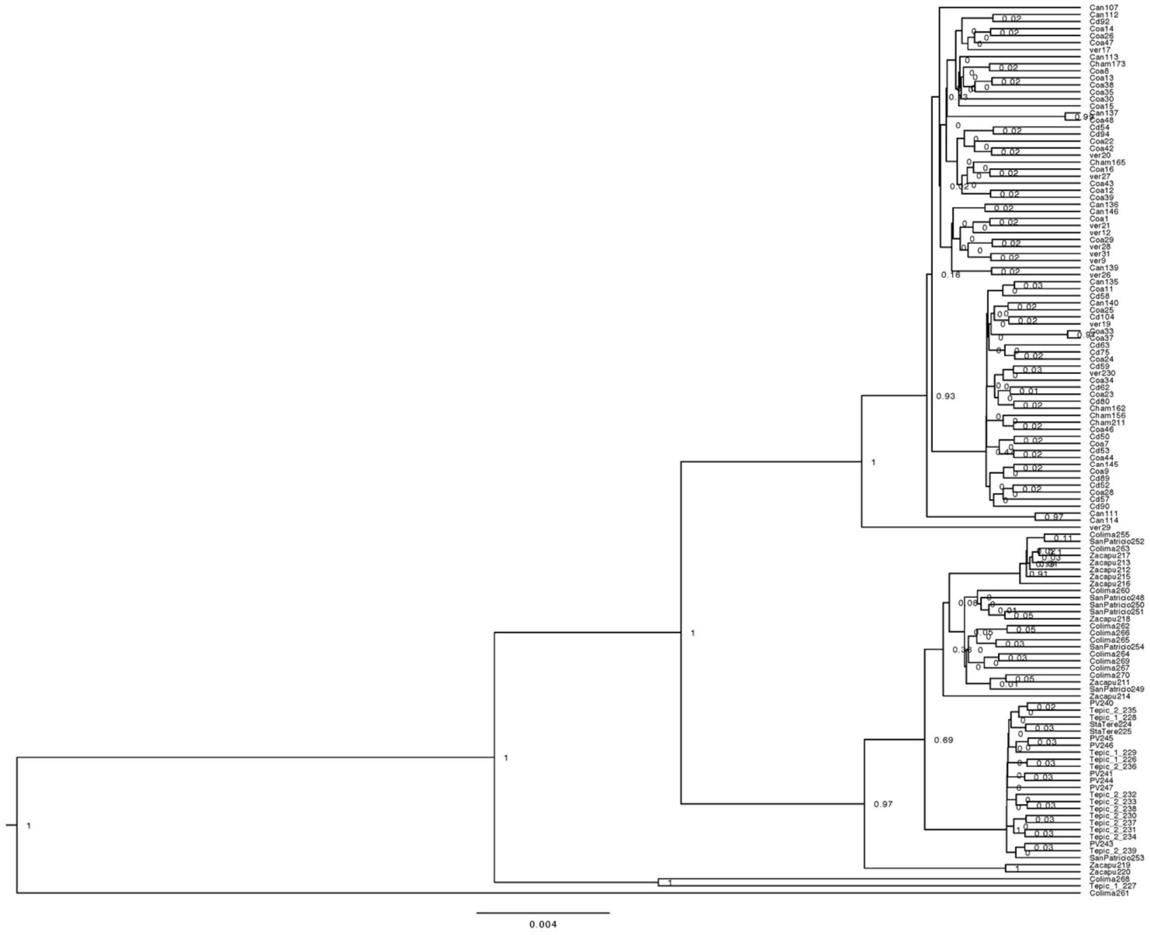

Figure S18. Bayesian phylogeny of *Membracis mexicana* using 28S gene segment.

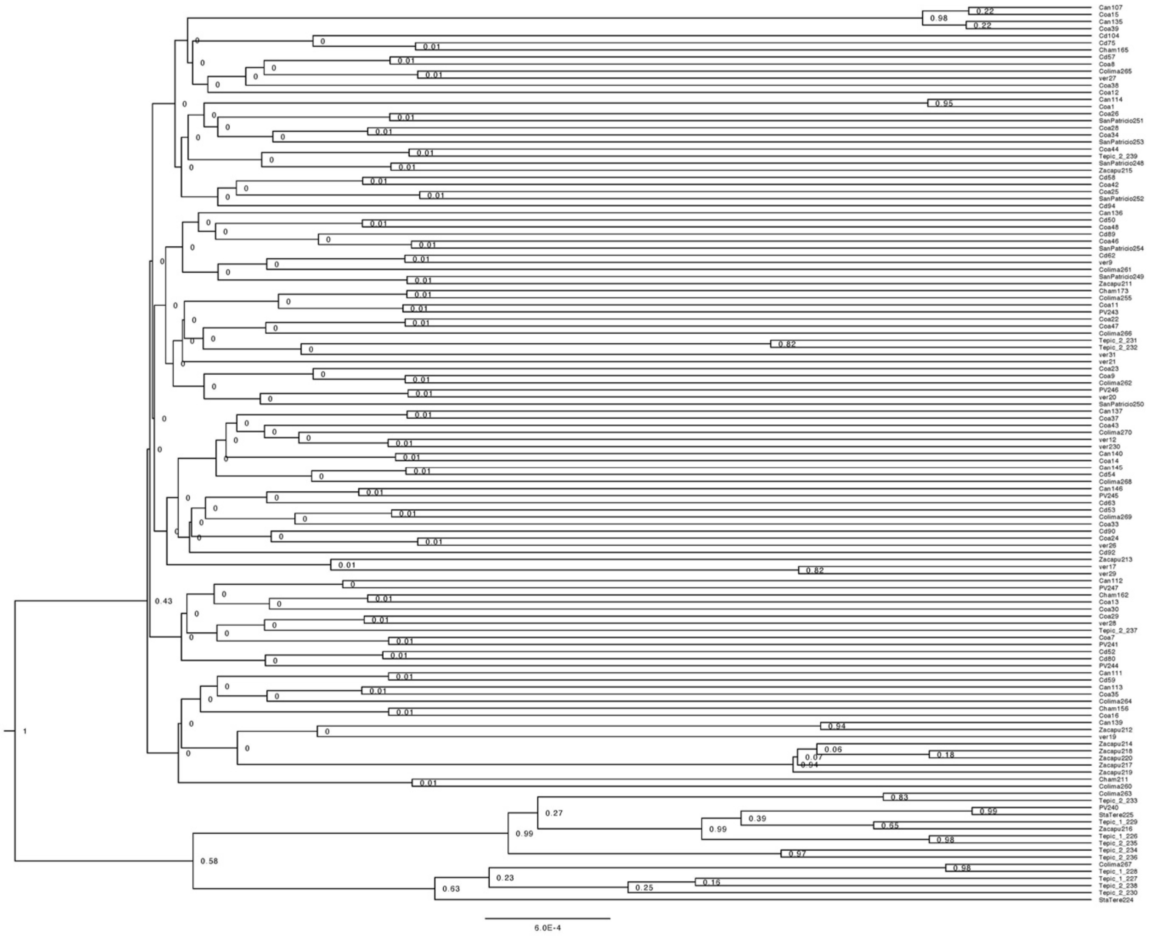

Figure S19. Bayesian phylogeny of *Membracis mexicana* using H2A gene segment.

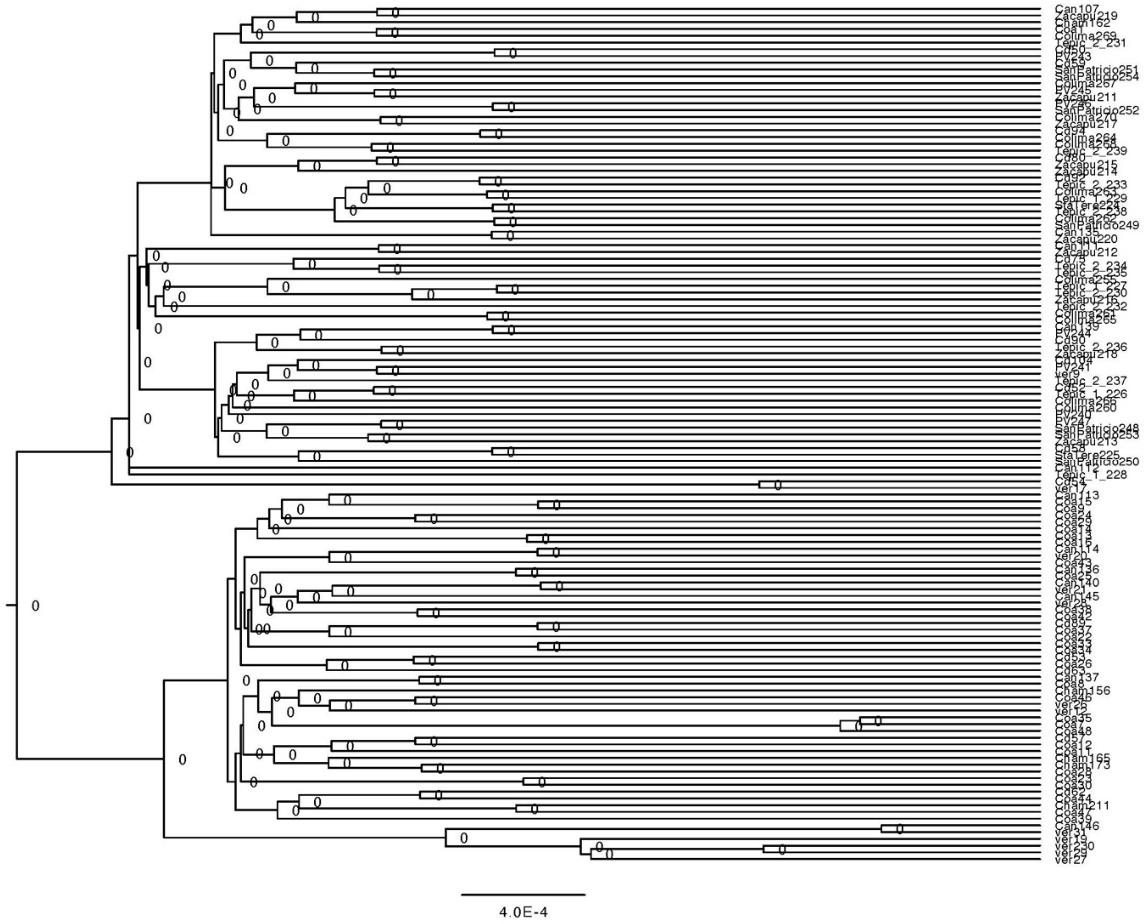

Figure S20. Bayesian phylogeny of *Membracis mexicana* using H3 gene segment.

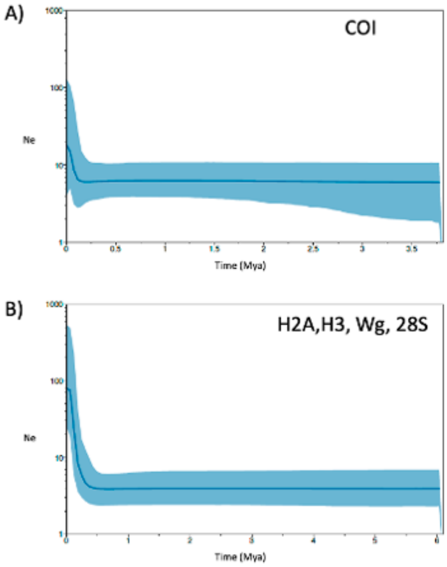

.
Figure S21. Skyline plot using mitochondrial (A) and nuclear markers (B) of *Membracis mexicana* from Mexico.
